# Exogenous melatonin reduces the inhibitory effect of osmotic stress on photosynthesis in soybean

**DOI:** 10.1371/journal.pone.0226542

**Published:** 2019-12-23

**Authors:** Mingcong Zhang, Songyu He, Yingce Zhan, Bin Qin, Xijun Jin, Mengxue Wang, Yuxian Zhang, Guohua Hu, Zhanlin Teng, Yaokun Wu

**Affiliations:** 1 College of Agronomy, Heilongjiang Bayi Agricultural University, Daqing, P.R. China; 2 Huanan Agrotechnical Extension Center, Jiamusi, P.R. China; 3 Daqing Branch of Heilongjiang Academy of Sciences, Daqing, P.R. China; Huazhong Agriculture University, CHINA

## Abstract

Understanding the relationship between exogenous melatonin and water deficit stress is crucial for achieving high yields and alleviating the effects of water deficit stress on soybean (*Glycine max (L*.*) Merrill*) plants in agriculture. This study investigated the effects of exogenous melatonin on soybean photosynthetic capacity under water deficit stress induced by polyethylene glycol (PEG) 6000. We conducted a potting experiment in 2018 using the soybean (*Glycine max* L. Merrill) cultivar Suinong 26. We identified the impacts of a concentration of PEG 6000 simulating drought (15%, w/v) and an appropriate melatonin concentration (100 μmol/L) on the growth of soybean seedlings and flowering stages in a preliminary test. We applied exogenous melatonin by foliar spraying and root application to determine the effects on leaf photosynthesis during water deficit stress. Our results indicated that 15% PEG 6000 had an obvious inhibitory effect on the growth of soybean seedlings and flowering stages, causing oxidative stress and damage due to reactive oxygen species (ROS) (H_2_O_2_ and O_2_^·-^) accumulation and potentially reducing air exchange parameters and photosystem II (PSII) efficiency. The application of exogenous melatonin significantly relieved the inhibitory effects of PEG 6000 stress on seedlings and flowering growth, and gas exchange parameters, potentially improved PSII efficiency, improved the leaf area index (LAI) and the accumulation of dry matter, slowed down oxidative stress and damage to leaves by increasing the activity of antioxidant enzymes (SOD, POD, and CAT), reduced the content of malondialdehyde (MDA), and ultimately improved soybean yield. Overall, the results of this study demonstrated that application of exogenous melatonin at the seedlings and flowering stages of soybean is effective in alleviating plant damage caused by water deficit stress and improving the drought resistance of soybean plants. In addition, the results showed that application of exogenous melatonin by root is superior to foliar spraying.

## Introduction

Drought is a major threat to plant growth and crop yields [1]. In the soybean (*Glycine max* L. Merrill) production process, water shortage directly reduces photosynthesis in leaves by affecting plant growth and development [2]. For example, the absorption of CO_2_ is limited by stomata and mesophyll organs [3]. In addition, under conditions of water deficit stress, the expansion of leaf cells is reduced, and the absorption of CO_2_ is limited to inhibit the growth of plants [4]. Excessive reactive oxygen species (ROS) are formed and rapidly accumulate in plants [5]. ROS (H_2_O_2_ and O_2_^·-^) inhibit leaf cell wall enzymes to affect the enzymatic activity of leaf photosynthesis [6]. The tolerance of plants to water deficit varies among different crops and varieties [7].

Water deficit stress reduces net photosynthetic rates in leaves by reducing nonstomatal factors such as ribulose bisphosphate carboxylase (Rubisco) and ribulose-1,5-bisphosphate (RuBP) [8]. Some crops close their stomata to reduce plant damage under drought conditions [9], which reduces the amount of CO_2_ absorption to decrease the photosynthetic capacity of mesophyll organs [10]. However, plants can adapt their photosynthetic metabolism upon application of exogenous hormones to survive under stress [11]. It has been reported that under water deficit stress, compared with no treatment, the application of exogenous hormones can significantly alleviate growth and developmental impairments in soybean and improve the activity of antioxidant enzymes [12] to enhance the ability of endogenous antioxidants to remove excessive ROS [8, 13].

Chlorophyll fluorescence (ChlF) is used to evaluate the integrity and efficiency of photosynthetic organs as well as the overall health of plant tissues [14]. Photosynthetic organs, especially PSII, are sensitive to water stress [15–16]. ChlF emission changes, which are mainly caused by PSII, provide information concerning almost all aspects of photosynthesis [17–18] and reflect a plant’s tolerance to environmental stresses, including drought. Fluorescence parameters have been used to identify leaf damage in the absence of obvious symptoms [18], and ChlF is often used as a potential index of environmental stress and a screening method for disease-resistant plants [19]. Fluorescence efficiency can be described by the optimal/maximal quantum yield (Fv/Fm), nonphotochemical quenching (NPQ), electron transport rate (ETR) and actual photochemical quantum yield (Φ_PSII_) [20]. Studies have shown that the ChlF parameter can be used to screen drought-tolerant germplasm resources of soybean and to evaluate the degree of drought stress present in soybean [21].

**Fig 1:**
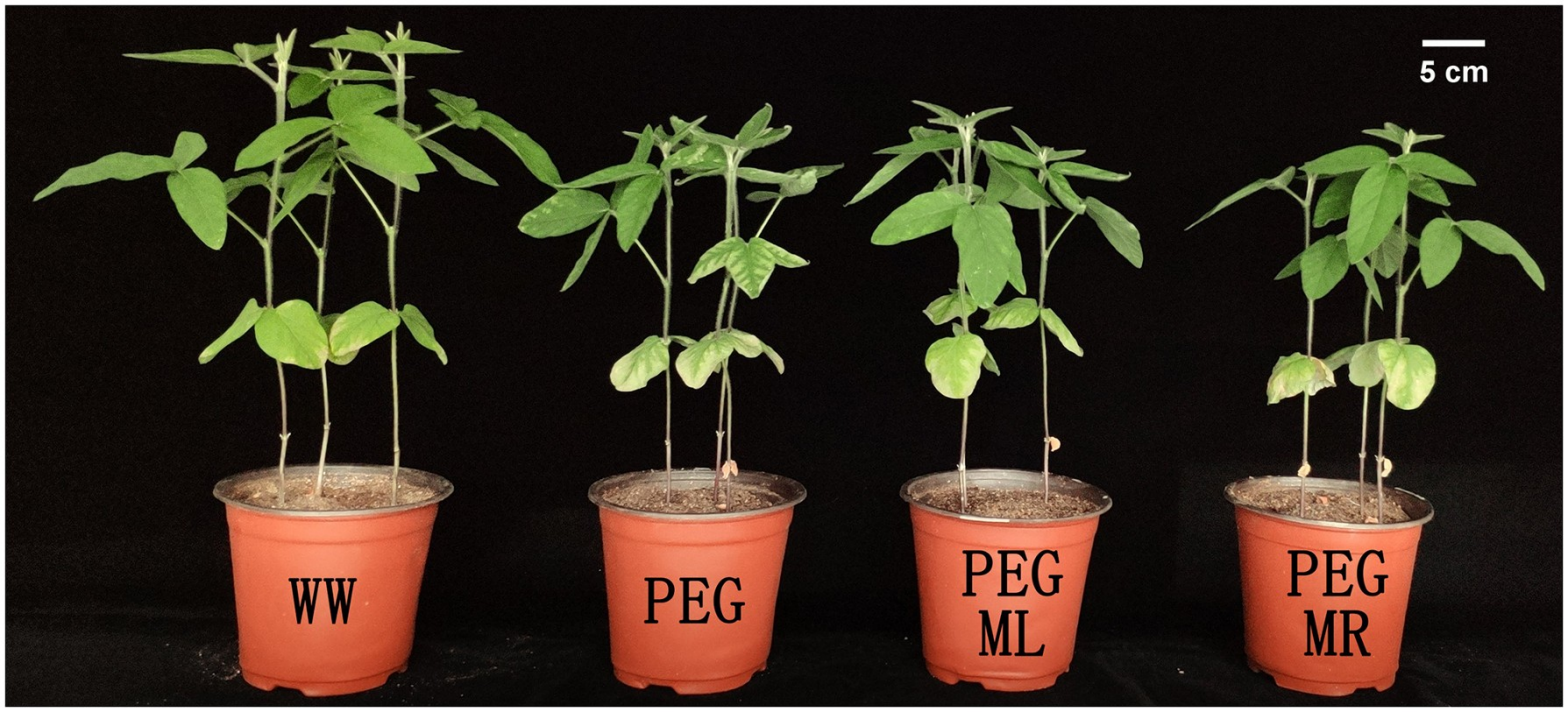
Effect of melatonin addition on the growth of soybean plant in our preliminary experiment. WW: half-strength Hoagland’s nutrient solution alone; PEG: half-strength Hoagland’s nutrient solution plus 15% PEG 6000 treatment; PEG+ML: half-strength Hoagland’s nutrient solution plus 15% PEG 6000 treatment and 100 μmol/L melatonin by leaf; PEG+MR: half-strength Hoagland’s nutrient solution plus 15% PEG 6000 treatment and 100 μmol/L melatonin by root.

It is difficult to control watering to reduce soil moisture at the vegetative or reproductive growth stage of soybean with drought stress. Li [22] suggested that vermiculite and perlite be used to replace agricultural soil, and polyethylene glycol (PEG) with a molecular weight of 6,000 was used to simulate drought stress in plants at any stage of soybean growth. PEG 6000 is an inert substance that cannot enter cell wall tissues, and it has been demonstrated that crops have difficulty absorbing PEG with a molecular weight exceeding 3000 [23]. As reported, PEG 6000 causes obvious water stress to plants without any toxic effects [24]. Our preliminary experiment [25] confirmed that a PEG 6000 concentration of 15% (w/v) had a strong water deficit stress effect on Suinong 26 at V3 stage; however, application of the appropriate melatonin concentration (100 μmol/L) was effective for alleviating plant damage caused by water deficit stress (Fig 1).

Melatonin (*N*-acetyl-5-methoxytryptamine), also known as the pineal hormone [26], was first detected in plants by Hattori [27–28]. Subsequent studies have detected melatonin in seeds, roots, fruits and leaves of plants [29–30]. One important function of melatonin is as an antioxidant that can alleviate plant damage under adverse conditions. Such antioxidant effects have been reported in several plants (e.g., apple [31], maize [32], wheat [33] and cucumber [34]). Research has shown that under water stress, melatonin can directly scavenge ROS and also improve the activity of antioxidant enzymes to alleviate the oxidative damage caused by water stress in cells [35]. Recent research results have suggested that melatonin can improve plant photosynthetic rates under water stress [36], inhibit chlorophyll degradation, and increase leaf photosynthetic rates and the accumulation of dry matter [37]. Previous studies have mainly focused on the beneficial effects of melatonin on plant stress resistance in maize [38], rice [39] and horticultural [3] crops, while few reports have investigated the effect of exogenous melatonin on photosynthetic reactions of soybean seedlings and flowering stages under water stress or on the adaptation of photosynthetic organs to water stress.

Therefore, the purpose of this study was to clarify the adaptive mechanism of soybean seedlings and flowering stages to water stress under exogenous melatonin to provide a theoretical basis for improving the drought resistance of soybean seedlings and flowering stages in actual production. The mechanism was clarified by analyzing the photosynthetic rates and fluorescence parameters of soybean at the V1, V3 and flowering stages under PEG 6000 stress.

## Materials and methods

### Test material

The soybean variety Suinong 26 was used as the test material in this experiment. This variety features infinite podding and a growth period of approximately 120 d. Melatonin was purchased from Sigma-Aldrich (St. Louis, MO, USA); the molecular formula of melatonin is C_13_H_16_N_2_O_2_, and the relative molecular mass is 232.28.

### Test method

The following experiments were conducted between May and October 2018 at the rainproof greenhouse of the National Engineering and Technology Research Center for Grains in the High-tech Zone, Daqing City, Heilongjiang Province, China (latitude 45°46' to 46°55', longitude 124°19' to 125°12', elevation 146 m). The experiment adopted a potting method. The pots were 36 cm tall with a diameter of 29 cm. On the bottoms of the pots, 5 holes (0.5 cm diameter) were drilled, and gauze was applied. Vermiculite and perlite were mixed 1:1 to serve as the culture medium. Full seeds with uniform colors and sizes were selected and disinfected with 5% sodium hypochlorite before they were sown on May 18. Nine seeds were sown in each pot. During the seedling period, 3 seedlings were consistently maintained evenly in each pot. The ripe seeds were harvested on October 2.

### Experimental treatments

The water deficit stress treatment was carried out at the V1 (first unrolled trifoliate leaf and June 4), V3(third unrolled trifoliate leaf and June 17) and R1 (beginning of the flowering stage, fifth unrolled trifoliate leaf and June 30) stages of soybean in this test, and the experimental plants were divided into six groups as follows: (i) the well-watered (WW) group, which was watered with 1 L of half-strength Hoagland’s nutrient solution every 3 d during the whole growth period (water leaked out of the bottom); (ii) the well-watered + melatonin by leaf (WW+ML) group, which received half-strength Hoagland’s nutrient solution and was treated with 100 μmol/L melatonin by leaf spraying (on the top and bottom sides of all leaves until water dripped); (iii) the well-watered + melatonin by root irrigation (WW+MR) group, which received half-strength Hoagland’s nutrient solution and was treated with 100 μmol/L melatonin by root; (iv) the drought stress (PEG) group, which received water deficit stress treatment with half-strength Hoagland’s nutrient solution plus 150 g of PEG 6000 (15%, w/v) (drought; water potential of -0.30 MPa); (v) the drought stress + melatonin by leaf (PEG+ML) group, which received half-strength Hoagland’s nutrient solution plus 15% PEG 6000 and was treated with 100 μmol/L melatonin by leaf spraying (on the top and bottom sides of all leaves until water dripped); and (vi) the drought stress + melatonin by root irrigation (PEG+MR) group, which received half-strength Hoagland’s nutrient solution plus 15% PEG 6000 and was treated with 100 μmol/L melatonin by root (in the preliminary test, it had already been demonstrated that 100 μmol/L melatonin had an ameliorative effect on soybean under water deficit stress). The water deficit stress treatment was conducted on day 0 and day 3 of the V1, V3 and R1 stages of soybean growth by applying half-strength Hoagland’s nutrient solution with 15% PEG 6000. Sampling and photosynthetic parameter determination were conducted at 9:00 am on the 6th day (nutrient solution not applied) after treatment of the V1, V3 and R1 stages. On the evening of the sampling day of sampling, clean water was used to flush the residual PEG 6000 until the solution was consistent with the WW solution, and 1/2 Hoagland’s nutrient solution was normally applied in the later stage to ensure normal growth and development.

### Testing items and methods

#### Leaf area index (LAI) analysis

Altogether, 5 pots of soybean were selected at random. A LI-3100C area meter (LiCor, Lincoln, NE) was used to measure the areas of all the leaves.

#### Leaf area duration (LAD) analysis

LAD (m^2^·d·m^-2^) = [(LA_1_+LA_2_)/2] ×(t_2_-t_1_), LA_1_ and LA_2_ represent the former and latter measurements of leaf area, and t_1_ and t_2_ represent the times at the former and latter points.

#### Dry matter accumulation analysis

Different soybean organs (e.g., leaves, petioles, stems, and roots) were subjected to 105 °C treatment for 30 min and dried at 70 °C to a constant weight, and the biomasses of the different parts of the plants were weighed. Each treatment had 5 replicates.

#### Determination of antioxidant enzyme activity and membrane lipid peroxidation

Five pots of soybean were randomly selected, and fully grown 2nd leaves were taken from each plant, wrapped in foil, and stored at -80°C. The activity of superoxide dismutase (SOD) was determined by using nitroblue tetrazole (NBT) method described by Beauchamp et al.[40], the activity of peroxidase (POD) was determined by using the guaiacol method described by Rao et al.[41], the activity of catalase (CAT) was determined by using the hydrogen peroxide method described by Hamurcu et al.[42], and the levels of malondialdehyde (MDA) were determined by the thiobarbituric acid method described by Draper et al.[43].

The superoxide anion production rate was determined. Briefly, 0.5 g of leaf tissue was ground into 10 ml of prechilled PBS (pH 7.8), and each homogenate was centrifuged at 13,000 x *g* for 20 minutes at 4°C. For the following analysis, 0.5 ml of the supernatant was added to 0.5 ml of PBS and 1 ml of 10 mmol/L hydroxylamine hydrochloride. After 1 hour, 2 ml of ether and 1 ml of 7 mmol α-naphthylamine were added at 4°C, and the mixtures were centrifuged at 3,000 x *g* for 20 minutes. The absorbance was measured at 530 nm using 0.5 ml of PBS as a reference blank according to the methods of Ke [44]. Each treatment was repeated 5 times.

#### Histochemical detection of H_2_O_2_ and O_2_^·-^ in soybean leaves

Detection of hydrogen peroxide (H_2_O_**2**_) in soybean leaves was performed by 3,3-diaminobenzidine (DAB) staining, and detection of superoxide radical (O_2_^·-^) was performed by NBT staining [45]. Soybean leaves were immersed and infiltrated under vacuum with 1.25 mg/mL DAB staining solution (pH 7.8) dissolved in H_2_O for 6 h and with 3 mg/mL nitroblue tetrazolium (NBT) staining solution in 10 mM potassium phosphate buffer (pH 7.0) containing 10 mM NaN_3_ for 30 min at room temperature. The stained leaves were bleached in an acetic acid: glycerol: ethanol (1:1:3 v/v) solution at 100°C for 5 min and stored in a glycerol: ethanol (1:4 v/v) solution until photographed.

#### Measurement of gas exchange parameters

The net photosynthetic rate (Pn), stomatal conductance (Gs), transpiration rate (Tr), and intercellular carbon dioxide concentration (Ci) of the middle part of the middle lobule of the 2^nd^ fully unfolded functional compound leaf from the main stem of each soybean plant were measured using a Li-6400 portable photosynthetic assay system (LICOR Inc., USA) between 09:00 and 11:30. The light source was an internally installed red-blue light, the plants were exposed to a constant photosynthetically active radiation of 1,200 μmol/m^2^/s, the air velocity was 500 mol/s, the CO_2_ supply concentration was 400 μmol/mol, the leaf chamber temperature was 25°C, the relative humidity was approximately 25% [46], and each treatment had 5 replicates.

#### Chlorophyll fluorescence

Between 09:00 and 11:30 on the date of sampling, an portable chlorophyll fluorometer (FMS-2, Hansatech, Englang) was used to detect the chlorophyll fluorescence parameters of the middle part of the middle lobule of the 2^nd^ fully unfolded functional compound leaf from the main stem of each soybean plant. Before measurement, the leaves were dark-adapted for 20 min; the minimum fluorescence under dark adaptation (Fo) was measured with low light, and then the maximum fluorescence (Fm) was measured with a saturating light pulse. When the fluorescence dropped from Fm to nearly Fo, the steady-state fluorescence (Fs) was measured with continuous actinic light. Later, a beam of saturating light was overlaid to detect the maximum fluorescence (Fm') under light. Finally, the actinic light was stopped, and far-red light was immediately turned on to measure the minimum fluorescence (Fo') under light and the electron transfer rate (ETR).

According to calculations based on Baker’s [46] chlorophyll fluorescence kinetic curve, variable fluorescence (Fv) = Fm–Fo, PSII primary light energy conversion efficiency (Fv/Fm) = (Fm-Fo)/Fm, the PSII photochemical quantum yield (Φ_PSII_) = (Fm'–Fs)/Fm', and the nonphotochemical quenching coefficient (NPQ) = Fm/Fm'-1. Five plants were measured under each treatment method, and each leaf was recorded three times. The average value was taken as the value of the photosynthetic parameter.

#### Yield and yield composition

Sampling were conducted at harvest time. Five pots of soybean under each treatment were randomly selected for assessment of plant height, number of pods per plant, total number of pods, and hundred-grain weight.

## Statistical analysis

Analysis of variance was performed by using Microsoft Office Excel 2019. The data are reported as the mean and standard error (SE) values of triplicate experiments and were analyzed by using SPSS 17.0. Function plots were constructed using Origin 2018. One-way analysis of variance (ANOVA) and Duncan’s multiple range tests were used to determine the significance of the differences among samples with a significance level of 0.05 or 0.01.

## Results

### Effect of exogenous melatonin on individual LAI and LAD values of soybean under osmotic stress

According to Table 1, LAI and LAD in stages V1, V3 and R1 showed no significant difference between the well-watered with melatonin (WW+ML and WW+MR) and the simple well-watered (WW) application; however, the PEG-simulated water stress treatments (PEG, PEG+ML, and PEG+MR) significantly reduced the LAI per plant in the seedling and flowering stages, and the LAI per plant values under V1, V3 and R1 water stress were significantly lower than under the WW condition. The amplitude was decreased by 19.7%~22.8% (*P* < 0.01) at the V1 stage, 26.0%~37.8% (*P* < 0.01) at the V3 stage and 36.3%~47.3% (P < 0.01) at the R1 stage. Application of exogenous melatonin could increase the LAI per plant in the soybean seedling stage. The LAI per plant values were higher in the groups treated with melatonin (PEG+ML and PEG+MR) under water stress at the V1 stage than in the groups without melatonin treatment (PEG), but the difference did not reach significance (5%). In stage V3, compared with PEG treatment, melatonin treatment by root irrigation (PEG+MR) increased the LAI per plant by 9.37% (*P*<0.05), while melatonin treatment by foliar spraying (PEG+ML) increased the value by 3.45% under water stress; this difference, however, did not reach significance. Additionally, the LAI per plant values were higher with the application of exogenous melatonin treatment (PEG+ML and PEG+MR) under water stress at the R1 stage than without melatonin treatment (PEG), with increases by 5.24% and 8.07% (*P*<0.05), respectively. Water stress markedly reduced the LAD of leaves at the V1-V3 and R1-V3 stages. Compared with the WW condition, PEG treatment decreased the LAD by 56.3% and 69.0% (*P*<0.05), respectively, and the application of exogenous melatonin increased the LAD of soybean leaves at the V1-V3 and R1-V3 stages under water stress. Compared with PEG, PEG+ML and PEG+MR increased the LAD by 4.68% and 4.90% (*P*<0.05) in the V1-V3 stage, and PEG+MR increased the LAD by 5.04% (*P*<0.05) in the R1-V3 stage.

**Table 1:** Effect of exogenous melatonin on individual LAI and LAD values of soybean under osmotic stress.

Treatment	Leaf area index (LAI) per plant			Leaf area duration (LAD) (m^2^·d·m^-2^)	
	V1	V3	R1	V3-V1	R1-V3
**WW**	0.0766±0.0009 aA	0.1560±0.0015 aA	0.2390±0.0019 aA	1.0018±0.0020 aA	1.1167±0.0251 aA
**WW+ML**	0.0773±0.0004 aA	0.1559±0.0006 aA	0.2400±0.0020 aA	0.9929±0.0108 aA	1.1310±0.0343 aA
**WW+MR**	0.0772±0.0003 aA	0.1563±0.0007 aA	0.2386±0.0035 aA	0.9986±0.0036 aA	1.1071±0.0487 aA
**PEG**	0.0624±0.0014 bB	0.1132±0.0024 cB	0.1623±0.0012 cB	0.6411±0.0062 cB	0.6608±0.0164 cB
**PEG+ML**	0.0636±0.0009 bB	0.1171±0.0026 bcB	0.1708±0.0011 bB	0.6711±0.0051 bB	0.6862±0.0146 cB
**PEG+MR**	0.0640±0.0015 bB	0.1238±0.0008 BB	0.1754±0.0012 bB	0.6725±0.0042 bB	0.6941±0.0184 bB

WW: half-strength Hoagland’s nutrient solution alone; WW+ML: half-strength Hoagland’s nutrient solution and 100 μmol/L melatonin by leaf; WW+MR: half-strength Hoagland’s nutrient solution and 100 μmol/L melatonin by root; PEG: half-strength Hoagland’s nutrient solution plus 15% PEG 6000 treatment; PEG+ML: half-strength Hoagland’s nutrient solution plus 15% PEG 6000 treatment and 100 μmol/L melatonin by leaf; PEG+MR: half-strength Hoagland’s nutrient solution plus 15% PEG 6000 treatment and 100 μmol/L melatonin by root. The data represent the mean ± SE of five replicate samples. Different lowercase letters indicate significant differences at *P*<0.05 level; capital letters indicate significant differences at *P*<0.01.

### Effect of exogenous melatonin on dry matter accumulation of soybean under osmotic stress

The accumulation of dry matter in various organs showed no significant difference between the well-watered with melatonin application (WW+ML and WW+MR) and simple well-watered (WW) groups (Table 2); however, compared with the WW treatment at the V1, V3 and R1 stages, the accumulation of dry matter in various organs was significantly reduced under water stress (PEG, PEG+ML, and PEG+MR). Compared with the WW treatment, PEG treatment in the V1 stage decreased dry matter accumulation in leaves, petioles, stems and roots by 14.3%, 60.0%, 42.9% and 32.0% (*P*<0.01), respectively; however, there was no significant difference between water deficit stress with melatonin application (PEG+ML and PEG+MR) and simple water deficit stress (PEG). The trend of water stress (PEG, PEG+ML, and PEG+MR) and WW treatment in stage V3 was equivalent to stage V1, but the trend between plants subjected to water stress with melatonin application (PEG+ML and PEG+MR) and to water stress alone (PEG) in stage V3 differed from those in stage V1. The accumulation of dry matter in leaves under water deficit stress treated with melatonin by leaf spraying (PEG+ML) was 9.09% (*P*<0.05) higher than in leaves under PEG treatment alone, and the accumulation of dry matter in roots under water deficit stress and treatment with melatonin by root irrigation (PEG+MR) was 17.3% (*P*<0.05) higher than in roots under PEG alone in stage V3. Compared with PEG treatment in stage R1, the accumulation of dry matter in leaves under water deficit stress and treated with melatonin by root irrigation (PEG+MR) was 9.48% (*P*<0.05) higher than in leaves under PEG treatment alone.

**Table 2:** Effect of exogenous melatonin on dry matter accumulation under osmotic stress.

Stage	Treatment	Leaves (g)	Petioles (g)	Stems (g)	Roots (g)
**V1**	**WW**	0.40±0.02aA	0.08±0.01abAB	0.20±0.02aA	0.66±0.02aA
	**WW+ML**	0.41±0.01aA	0.09±0.01aA	0.19±0.02aAB	0.68±0.02aA
	**WW+MR**	0.39±0.02abA	0.09±0.02aA	0.18±0.02abAB	0.66±0.01aA
	**PEG**	0.35±0.03cB	0.05±0.01cB	0.14±0.01cC	0.50±0.03cC
	**PEG+ML**	0.35±0.05cB	0.05±0.01cB	0.14±0.01cC	0.56±0.04bcBC
	**PEG+MR**	0.36±0.02bcAB	0.06±0.01bcAB	0.15±0.02bcBC	0.59±0.03bB
**V3**	**WW**	0.52±0.01aA	0.15±0.02aA	0.31±0.01aA	1.01±0.02aA
	**WW+ML**	0.53±0.02aA	0.17±0.01aA	0.33±0.03aA	1.02±0.01aA
	**WW+MR**	0.55±0.03aA	0.16±0.02aA	0.32±0.02aA	1.00±0.03aA
	**PEG**	0.44±0.01cA	0.12±0.01bA	0.25±0.01bA	0.81±0.02cB
	**PEG+ML**	0.48±0.02bA	0.13±0.01bA	0.27±0.01bA	0.90±0.03bcAB
	**PEG+MR**	0.46±0.01bcA	0.12±0.02bA	0.27±0.01bA	0.95±0.03bAB
**R1**	**WW**	2.39±0.20aA	0.89±0.11aA	1.21±0.06aA	2.03±0.06aA
	**WW+ML**	2.42±0.22aA	0.91±0.09aA	1.19±0.06aA	2.06±0.11aA
	**WW+MR**	2.38±0.19aA	0.88±0.12aA	1.20±0.09aA	2.11±0.16aA
	**PEG**	1.69±0.11cB	0.64±0.02bB	0.83±0.05bB	1.80±0.09bB
	**PEG+ML**	1.81±0.16bcAB	0.69±0.10bAB	0.87±0.06bB	1.86±0.08bAB
	**PEG+MR**	1.85±0.10bAB	0.71±0.09bAB	0.88±0.03bB	1.91±0.10abAB

WW: half-strength Hoagland’s nutrient solution alone; WW+ML: half-strength Hoagland’s nutrient solution and 100 μmol/L melatonin by leaf; WW+MR: half-strength Hoagland’s nutrient solution and 100 μmol/L melatonin by root; PEG: half-strength Hoagland’s nutrient solution plus 15% PEG 6000 treatment; PEG+ML: half-strength Hoagland’s nutrient solution plus 15% PEG 6000 treatment and 100 μmol/L melatonin by leaf; PEG+MR: half-strength Hoagland’s nutrient solution plus 15% PEG 6000 treatment and 100 μmol/L melatonin by root. The data represent the mean ± SE of five replicate samples. Different lowercase letters and indicate significant differences at *P*<0.05 level; capital letters indicate significant differences at *P*<0.01.

### Effect of exogenous melatonin on antioxidant systems and MDA levels of soybean leaves under osmotic stress

**Fig 2:**
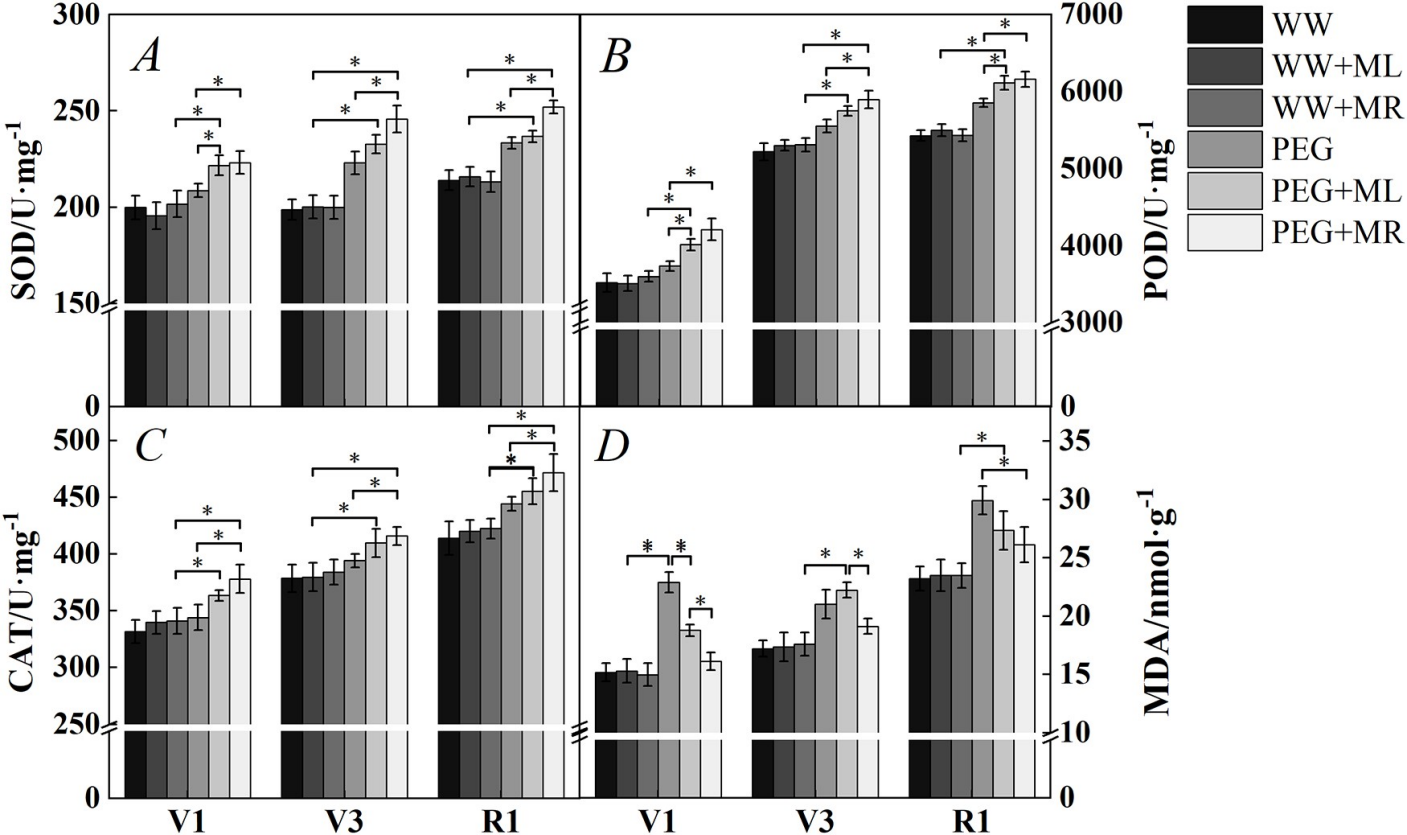
Effect of melatonin addition on the activity level of superoxide dismutase (SOD) (A), peroxidase (POD) (B), and catalase (CAT) (C) and the content of malondialdehyde (MDA) (D) under osmotic stress. WW: half-strength Hoagland’s nutrient solution alone; WW+ML: half-strength Hoagland’s nutrient solution and 100 μmol/L melatonin by leaf; WW+MR: half-strength Hoagland’s nutrient solution and 100 μmol/L melatonin by root; PEG: half-strength Hoagland’s nutrient solution plus 15% PEG 6000 treatment; PEG+ML: half-strength Hoagland’s nutrient solution plus 15% PEG 6000 treatment and 100 μmol/L melatonin by leaf; PEG+MR: half-strength Hoagland’s nutrient solution plus 15% PEG 6000 treatment and 100 μmol/L melatonin by root. The data represent the mean ± SE of five replicate samples. * indicate significant differences according to Duncan’s multiple range test (*P*<0.05).

The SOD activity of soybean leaves under water deficit stress (PEG, PEG+ML, and PEG+MR) was higher than in soybean leaves in the WW condition in stage V1 (Fig 2A), while the SOD activity under melatonin treatment and water deficit stress (PEG+ML and PEG+MR) was higher than under water deficit stress alone (PEG). The activity was 6.95% higher (*P*<0.05) in plants under PEG treatment with melatonin by root irrigation (PEG+MR) than in plants under PEG treatment alone. Stages V3 and R1 showed the same trend as Stage V1, but the activity under PEG+MR was 10.22% and 7.96% higher (*P*<0.05) than under PEG alone respectively, and thus, the rate of increase in stage V3 was greater compared with the other stages.

POD and CAT jointly eliminate H_2_O_2_ produced by SOD disproportionation reactions and maintain reactive oxygen at normal levels [47]. As shown in Fig 2B and 2C, POD and CAT activities were higher in soybean leaves in stage R1 than stages V1 and V3, and the results showed essentially the same trend as reported for stages V1, V3 and R1. POD and CAT activities were higher in soybean leaves under water stress (PEG, PEG+ML, and PEG+MR) than leaves under the WW condition, while they were higher under PEG and melatonin treatment than under PEG alone. The activities of both POD and CAT showed greater increases with melatonin application by root irrigation than by leaf spraying. The POD activity levels were increased with PEG+MR treatment in V1, V3 and R1 compared with PEG alone by 12.8%, 6.21% and 5.21%(*P*<0.05), and CAT was increased by 9.88%, 5.49% and 6.20%(*P*<0.05), respectively.

To some extent, the MDA content can reflect the degree of drought stress in plants. The higher the content, the more severe is the cell membrane lipid peroxidation [48]. As shown in Fig 2D, the MDA content of soybean leaves increased rapidly after water deficit stress. The content increased by 42.1%, 9.93% and 14.5% (*P*<0.05), respectively, in stages V1, V3 and R1 under PEG treatment compared with WW treatment; however, melatonin treatment (PEG+ML and PEG+MR) under water deficit stress reduced the MDA content. Compared with PEG, PEG+ML reduced the content by 21.9% in stage V1 and by 9.40% in stage R1 (*P*<0.05), and PEG+MR reduced the content by 42.1% in stage V1, by 9.93% in stage V3 and by 14.5% in stage R1 (*P*<0.05). Furthermore, PEG+MR induced a decrease by 16.5% in stage V1, 16.3% in stage V3 and 4.63% in stage R1 (*P*<0.05) compared with PEG+ML. These findings suggest that melatonin administration by root irrigation could better regulate the activity of antioxidant enzymes in soybean leaves and reduce the degree of damage as compared to administration by leaf spraying.

### Effect of exogenous melatonin on H_2_O_2_ contents and O_2_^·-^ production rates of soybean leaves under osmotic stress

**Fig 3:**
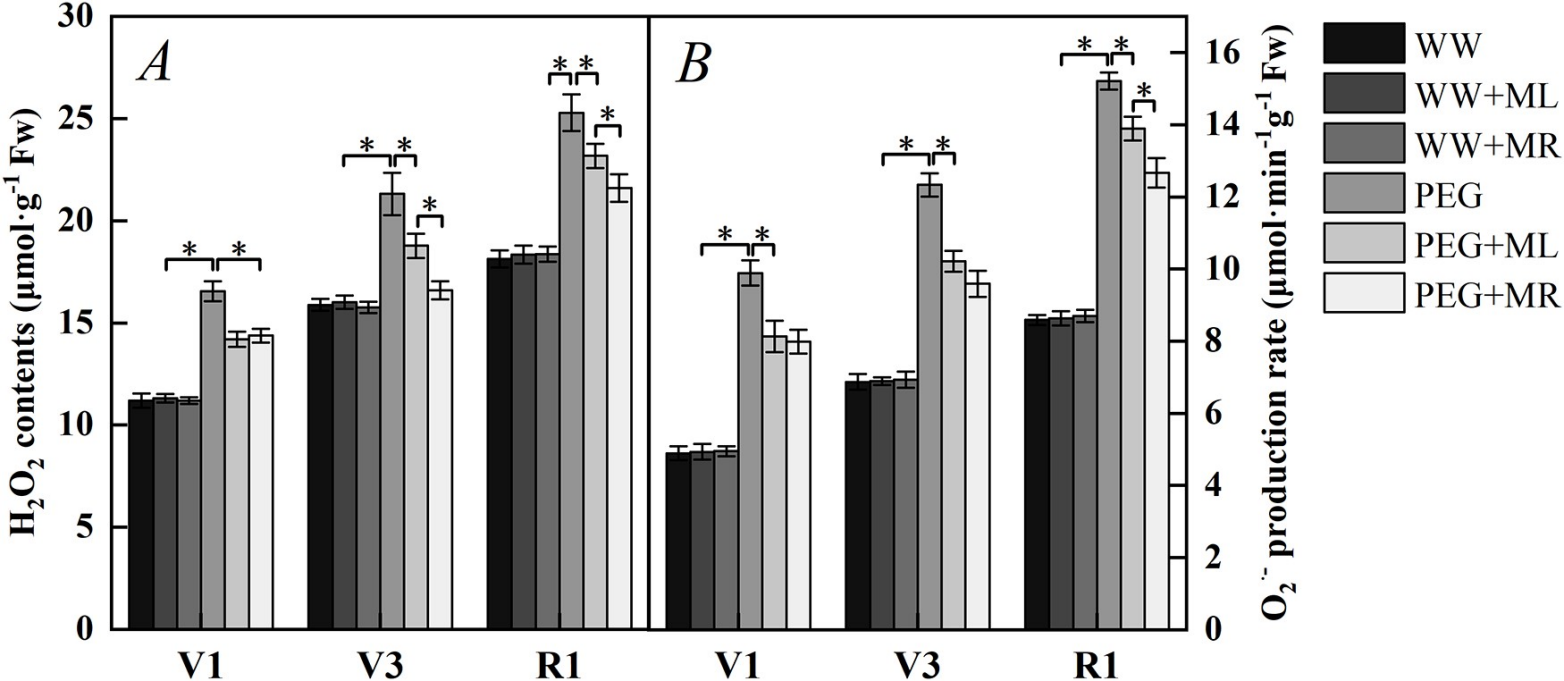
Effect of melatonin addition on hydrogen peroxide (H_2_O_2_) content (A) and superoxide anion (O2·-) production rates (B) of soybean leaves under osmotic stress. WW: half-strength Hoagland’s nutrient solution alone; WW+ML: half-strength Hoagland’s nutrient solution and 100 μmol/L melatonin by leaf; WW+MR: half-strength Hoagland’s nutrient solution and 100 μmol/L melatonin by root; PEG: half-strength Hoagland’s nutrient solution plus 15% PEG 6000 treatment; PEG+ML: half-strength Hoagland’s nutrient solution plus 15% PEG 6000 treatment and 100 μmol/L melatonin by leaf; PEG+MR: half-strength Hoagland’s nutrient solution plus 15% PEG 6000 treatment and 100 μmol/L melatonin by root. The data represent the mean ± SE of five replicate samples. * indicate significant differences according to Duncan’s multiple range test (*P*<0.05).

As shown in Fig 3(A) and 3(B), compared with the WW condition, water stress treatment significantly increased the ROS (H_2_O_2_ and O_2_^·-^) content in leaves, indicating that water deficit stress promoted the accumulation of anions in leaves, while melatonin treatment under water deficit stress significantly reduced the ROS content in leaves compared with PEG treatment alone. In stage V1, compared with PEG, PEG+ML and PEG+MR reduced H_2_O_2_ by 16.6% and 15.1% (*P*<0.05) (Fig 3A), and reduced O_2_^·-^ by 21.6% and 23.9% (*P*<0.05) (Fig 3B); compared with PEG in stage V3, PEG+ML and PEG+MR reduced H_2_O_2_ by 13.5% and 28.5%, and reduced O_2_^·-^ by 20.6% and 28.6% (*P*<0.05); compared with PEG in stage R1, PEG+ML and PEG+MR reduced H_2_O_2_ by 9.10% and 17.1%, and reduced O_2_^·-^ by 9.50% and 22.0% (*P*<0.05); respectively. This result indicates that melatonin can reduce ROS (H_2_O_2_ and O_2_^·-^) content in leaves under water deficit stress, alleviate ROS damage to plant cells in leaves, and maintain the balance of reactive oxygen in plant leaf tissues.

**Fig 4:**
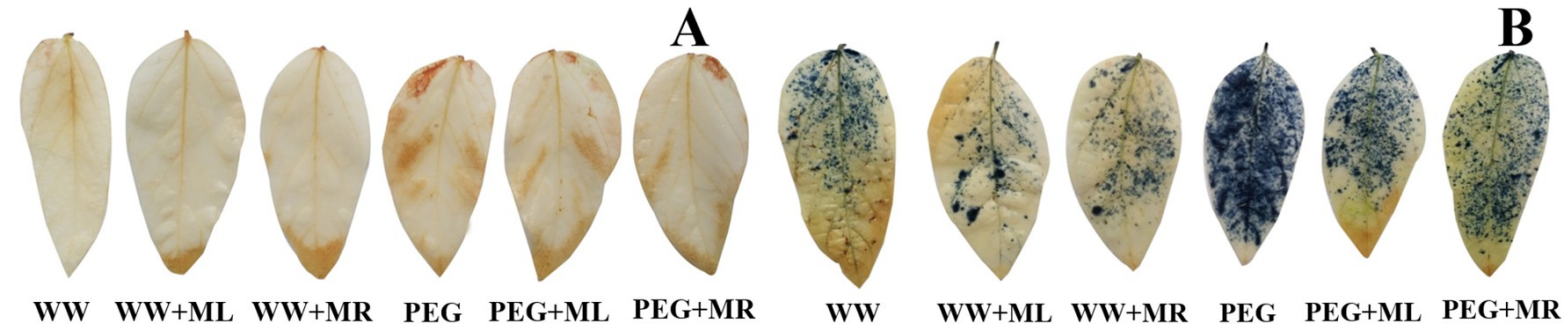
Effect of melatonin addition on histochemical localization of H_2_O_2_ (A) by the DAB uptake method and of O2·- (B) by NBT staining at V3 stage during water deficit stress treatment. WW: half-strength Hoagland’s nutrient solution alone; WW+ML: half-strength Hoagland’s nutrient solution and 100 μmol/L melatonin by leaf; WW+MR: half-strength Hoagland’s nutrient solution and 100 μmol/L melatonin by root; PEG: half-strength Hoagland’s nutrient solution plus 15% PEG 6000 treatment; PEG+ML: half-strength Hoagland’s nutrient solution plus 15% PEG 6000 treatment and 100 μmol/L melatonin by leaf; PEG+MR: half-strength Hoagland’s nutrient solution plus 15% PEG 6000 treatment and 100 μmol/L melatonin by root.

According to the method of Awasthi [45], H_2_O_2_ and O_2_^·-^ production in water deficit-stressed (PEG 6000; water potential of -0.30 MPa) and unstressed soybean seedling leaves was investigated qualitatively using DAB and NBT histochemical staining, respectively (Fig 4A and 4B). Under normal physiological water conditions, soybean seedling leaves showed low production of H_2_O_2_ and O_2_^·-^, and there was no significant different between the well-watered with melatonin application (WW+ML and WW+MR) and well-water alone (WW). However, soybean seedling leaves showed higher production under water deficit stress (PEG), while the leaves under PEG+ML and PEG+MR treatment exhibited markedly lower DAB and NBT staining, particularly compared with those under PEG treatment, which is an indication of less ROS production and less oxidative damage (Fig 4A and 4B).

### Effect of exogenous melatonin on fluorescence characteristics of soybean leaves under osmotic stress

**Fig 5:**
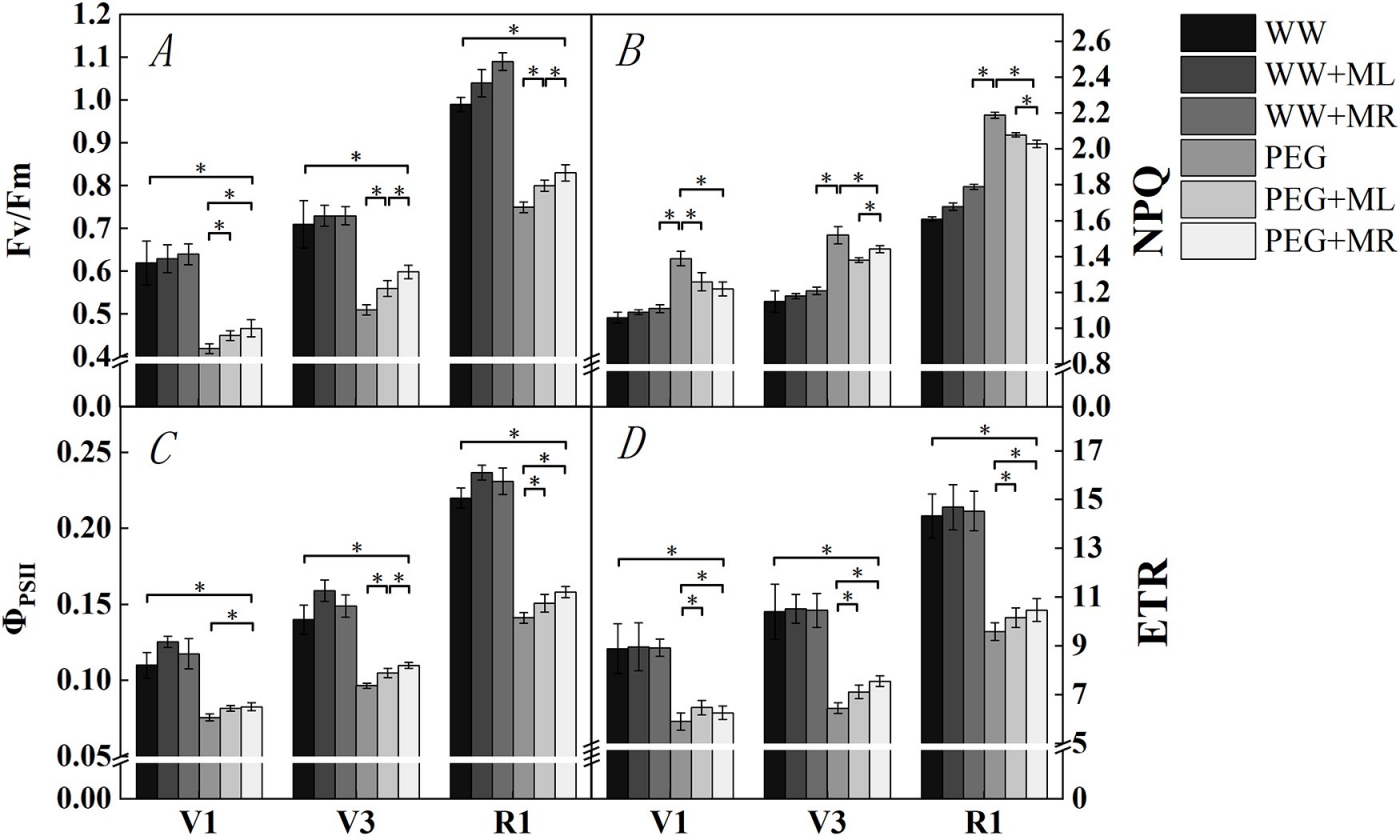
Effect of melatonin addition on PSII primary light energy conversion efficiency under osmotic stress. The PSII primary light energy conversion efficiency (Fv/Fm) (A), photochemical quenching coefficient (NPQ) (B), PSII actual photochemical quantum yield (Φ_PSII_) (C), and electron transfer rate (ETR) (D) of soybean leaves in seedlings and flowering stages during water deficit stress treatment. WW: half-strength Hoagland’s nutrient solution alone; WW+ML: half-strength Hoagland’s nutrient solution and 100 μmol/L melatonin by leaf; WW+MR: half-strength Hoagland’s nutrient solution and 100 μmol/L melatonin by root; PEG: half-strength Hoagland’s nutrient solution plus 15% PEG 6000 treatment; PEG+ML: half-strength Hoagland’s nutrient solution plus 15% PEG 6000treatment and 100 μmol/L melatonin by leaf; PEG+MR: half-strength Hoagland’s nutrient solution plus 15% PEG 6000 treatment and 100 μmol/L melatonin by root. The data represent the mean ± SE of five replicate samples. * indicate significant differences according to Duncan’s multiple range test (*P*<0.05).

Fig 5 shows that water deficit stress had an impact on the chlorophyll fluorescence parameters of soybean leaves in seedlings and flowering stages. Overall, the Fv/Fm, Φ_PS(II)_ and ETR of soybean seedlings and flowering stages demonstrated considerable downward trends under water deficit stress, while NPQ showed an upward trend. Compared with those under the WW condition, PSII under water deficit stress exhibited maximum decreases in Fv/Fm, ΦPS (II) and ETR of 47.6%, 45.5% and 50.7% (*P*<0.05) in stage V1, 39.2%, 44.9% and 61.2% (*P*<0.05) in stage V3, and 32.0%, 55.8 and 49.4% (*P*<0.05) in stage R1, respectively. NPQ increased by 31.1%, 32.2% and 36.0% (*P*<0.05) in V1, V3 and R1, respectively. The application of exogenous melatonin under PEG+ML and PEG+MR treatment increased, Fv/Fm, Φ_PS(II)_ and ETR to obviously higher levels than those under PEG treatment; the levels in the PEG+ML and PEG+MR groups increased by 7.17% and 11.2%, 7.93% and 9.39%, 9.76% and 6.03% (*P*<0.05) in V1, by 9.80% and 17.4%, 8.54% and 13.8%, 10.3% and 17.1% (*P*<0.05) in V3, and by 6.67% and 10.7%, 6.73% and 12.0%, 5.91% and 9.17 (*P*<0.05) in stage R1, respectively. There was no significant difference between the PEG+ML and PEG+MR groups under water deficit stress in stage V1, while PEG+MR treatment in stage V3 resulted in significantly higher values than PEG+ML, increasing the Fv/Fm, Φ_PS(II)_ and ETR by 6.91%, 4.82% and 6.18% (*P*<0.05), respectively, indicating that root application under water deficit stress has a better alleviating effect than foliar spray in stage V3.

NPQ reflects the heat dissipation of PSII-absorbed light energy in the process of photosynthetic electron transport [49]. The NPQ trend was contrary to the trend in Fv/Fm. PEG treatment resulted in a significantly higher NPQ than the WW condition. PEG+ML and PEG+MR reduced NPQ in soybean leaves under water deficit stress. Compared with PEG alone, the NPQ of PEG+ML and PEG+MR decreased by 9.35% and 12.2% in stage V1, by 9.21% and 5.13% in stage V3, and by 5.02% and 7.31% in stage R1, respectively, and the differences reached significance (5%).

### Effect of exogenous melatonin on gas exchange parameters of soybean leaves under osmotic stress

**Fig 6:**
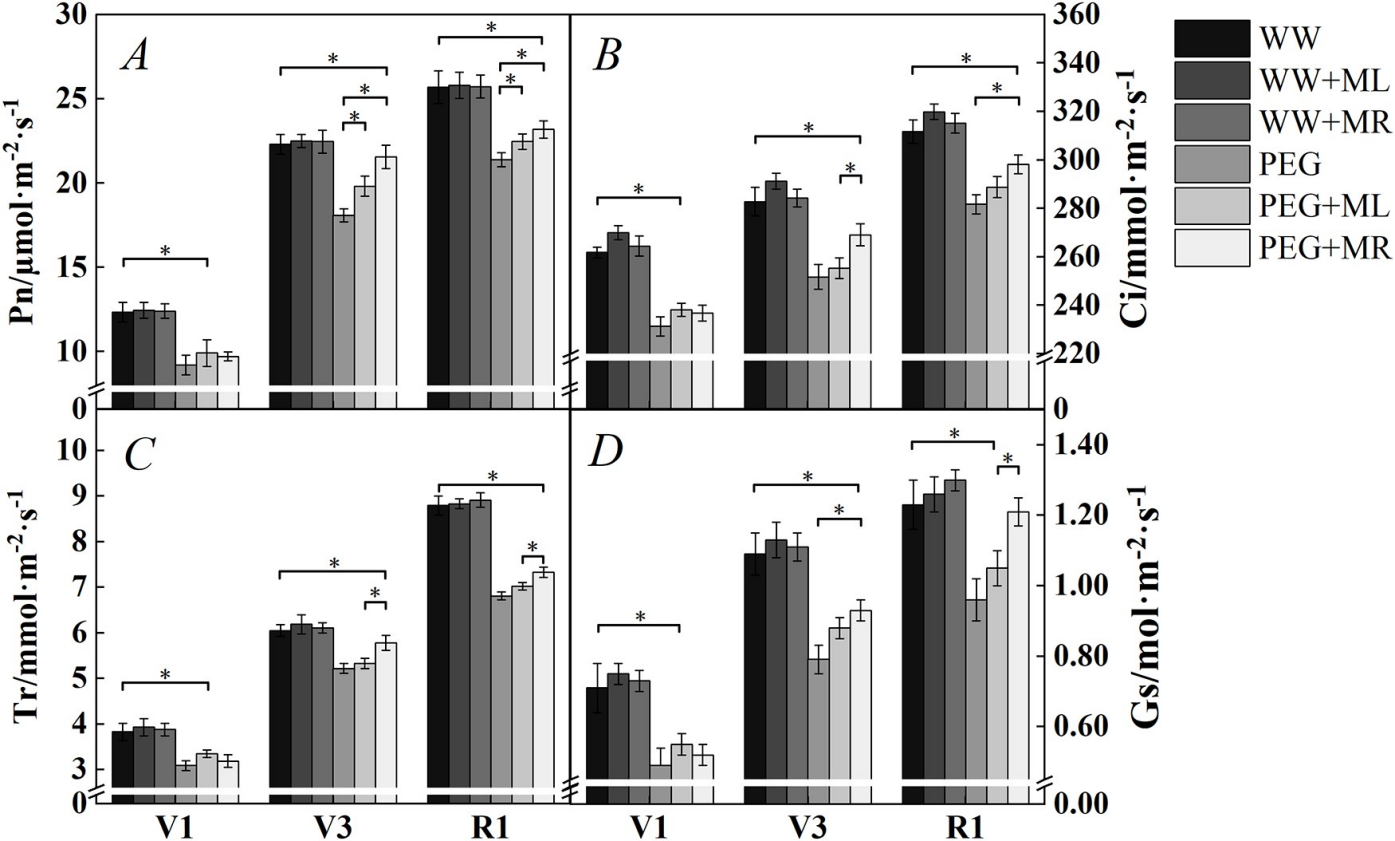
Effect of melatonin addition on the net photosynthetic rate under osmotic stress. The net photosynthetic rate (Pn) (A), intercellular CO_2_ concentration (Ci) (B), transpiration rate (Tr) (C), and stomatal conductance (Gs) (D) of soybean leaves in seedlings and flowering stages during water deficit stress treatment. WW: half-strength Hoagland’s nutrient solution alone; WW+ML: half-strength Hoagland’s nutrient solution and 100 μmol/L melatonin by leaf; WW+MR: half-strength Hoagland’s nutrient solution and 100 μmol/L melatonin by root; PEG: half-strength Hoagland’s nutrient solution plus 15% PEG 6000 treatment; PEG+ML: half-strength Hoagland’s nutrient solution plus 15% PEG 6000 treatment and 100 μmol/L melatonin by leaf; PEG+MR: half-strength Hoagland’s nutrient solution plus 15% PEG 6000 treatment and 100 μmol/L melatonin by root. The data represent the mean ± SE of five replicate samples. * indicate significant differences according to Duncan’s multiple range test (*P*<0.05).

Based on Fig 6, water deficit stress significantly reduced the net photosynthetic rate (Pn) (Fig 6A); however, application of exogenous melatonin increased the Pn. PEG+ML and PEG+MR in stages V3 and R1 increased the Pn by 9.56% and 19.1%, 5.00% and 8.42% (*P*<0.05), respectively, compared to PEG treatment alone. Under the influence of water deficit stress, the intercellular carbon dioxide concentration (Ci) (Fig 6B) in leaves also decreased significantly. However, the content of Ci under PEG+MR treatment in stage V3 and stage R1 compared with PEG treatment alone was increased by 6.91% and 5.86% (*P*<0.05), respectively.

Water deficit stress also significantly reduced stomatal conductance (Gs) (Fig 6D) in soybean leaves, and the decrease in Gs reduced stomatal activity, thus slowing transpiration and reducing the transpiration rate (Tr). The application of exogenous melatonin increased the Tr and Gs of soybean leaves under water deficit stress; the increases under PEG+MR treatment in stage V3 and stage R1 compared with PEG treatment were 10.7% (Fig 6C) and 17.6% (Fig 6D), and 7.64% and 26.1% (*P*<0.05), respectively. Thus, exogenous melatonin could alleviate the influence of water deficit stress on photosynthesis in soybean leaves by improving the Pn, Gs, Tr and Ci in soybean leaves and by maintaining strong photosynthesis to ensure the normal growth and development of soybean leaves.

### Effect of exogenous melatonin on soybean yield and yield composition under osmotic stress

As shown in Table 3, soybean yield and yield components in stages V1, V3 and R1 showed no significant difference between the well-watered with melatonin application (WW+ML and WW+MR) and simple well-watered (WW) groups. However, the soybean plant height was significantly reduced under water deficit stress. Compared with the WW condition, PEG treatment reduced the height in stages V1, V3 and R1 by 27.1%, 24.1% and 19.4% (*P*<0.05), respectively, indicating that water deficit stress had a great impact on plant height. However, plants under PEG+MR treatment in stages V1, V3 and R1 were significantly taller than those under PEG treatment alone, while PEG+MR treatment in stages V1 and R1 resulted in significantly higher values than PEG+ML, increasing the plant height by 6.18% and 8.59% (*P*<0.05), respectively. The numbers of soybean pods were also affected by water deficit stress; the numbers of 2-, 3- and 4-grain pods were significantly reduced under water stress but increased after concurrent application of exogenous melatonin, such that the total grain numbers of the plants were increased. Table 3 shows that compared with PEG, PEG+ML and PEG+MR increased the total numbers of grains by 29.1% and 39.0%, 27.4% and 26.4% (*P*<0.05), and 9.75% and 22.3% (*P*<0.05) in stages V1, V3 and R1, respectively. According to the yield results (Table 3), compared with PEG, PEG+ML and PEG+MR increased the yield by 31.6% and 43.3%, 29.8% and 31.8%, and 12.6% and 25.0% (*P*<0.05) in the V1, V3 and R1 stages, respectively, which indicated that PEG+MR was superior to PEG+ML.

**Table 3:** Effects of external melatonin on soybean yield and yield components.

Treatment		PH/cm	One SP	Two SP	Three SP	Four SP	SPP	100-SW/g	Yield (g/plant)
**V1**	**WW**	83.7±3.89a	3.71±0.59a	11.0±2.11a	13.6±1.35a	2.19±0.17a	75.3±2.35a	18.03±0.21a	13.57±0.59a
	**WW+ML**	84.9±2.88a	3.77±0.69a	10.89±1.01a	13.2±1.11a	2.31±0.19a	74.39±3.01a	18.11±0.11a	13.47±0.36a
	**WW+MR**	84.2±2.99a	3.80±0.31a	11.03±0.98a	13.2±1.21a	2.44±0.11a	75.22±2.84a	18.01±0.10a	13.62±0.59a
	**PEG**	61.0±5.87d	1.33±0.31c	6.17± 0.99c	9.50±0.89b	2.33±0.15b	51.5±3.19c	17.19±0.10a	8.85±1.01d
	**PEG+ML**	71.2±2.35c	2.33±0.39b	9.50±1.24b	12.8±1.28ab	1.67±0.09c	66.5±1.89b	17.52±0.11a	11.65±0.59c
	**PEG+MR**	75.60±1.11b	1.60±0.35c	9.20±1.07b	13.2±1.11a	3.00±0.13a	71.6±2.21ab	17.71±0.09a	12.68±0.99b
**V3**	**WW**	83.7±3.89a	3.71±0.39ab	11.0±2.11a	13.6±1.35a	2.19±0.17a	75.3±2.35a	18.03±0.21a	13.57±0.59a
	**WW+ML**	85.1±4.03a	3.48±0.29a	12.3±1.11a	12.98±0.89a	2.21±0.16a	75.86±1.36a	18.09±0.11a	13.72±0.29a
	**WW+MR**	84.5±4.38a	3.59±0.33a	10.98±1.39a	13.03±1.21a	2.23±0.19a	73.56±1.29a	18.02±0.10a	13.26±0.69a
	**PEG**	63.5±3.88c	2.25±0.19c	5.75±1.69b	10.5±1.27b	1.01±0.11b	49.3±1.89c	17.22±0.14a	8.48±1.03c
	**PEG+ML**	66.8±4.99bc	4.00±0.34a	10.8±1.89a	9.75±0.89c	2.02±0.08a	62.8±2.19b	17.55±0.10a	11.01±0.88b
	**PEG+MR**	72.8±5.69b	3.50±0.28b	5.50±1.37b	13.3±1.07a	2.05±0.11a	62.3±2.09b	17.96±0.07a	11.18±1.06b
**R1**	**WW**	83.7±3.89a	3.71±0.39a	11.0±2.11a	13.6±1.35a	2.19±0.17a	75.3±2.35a	18.03±0.21a	13.57±0.59a
	**WW+ML**	85.3±3.19a	3.75±0.21a	11.26±1.03a	12.88±1.02a	3.02±0.21a	76.99±0.16a	18.13±0.11a	13.96±0.36a
	**WW+MR**	84.9±2.99a	3.69±0.11a	11.33±0.65a	13.5±0.59a	2.29±0.13a	76.01±0.14a	18.1±0.09a	13.76±0.44a
	**PEG**	67.50±2.59c	1.50±0.13c	7.67±1.43d	10.50±0.89b	1.17±0.28b	53.00±2.13d	17.23±0.02c	9.13±0.99d
	**PEG+ML**	72.17±1.86b	2.33±0.23b	9.83±0.79b	10.50±1.35b	1.17±0.48b	58.17±2.09c	17.68±0.05b	10.28±1.03c
	**PEG+MR**	78.40±1.74a	2.40±0.65b	8.20±2.36c	12.40±1.36ab	2.20±0.49a	64.80±2.56b	17.61±0.02b	11.41±0.93b

WW: half-strength Hoagland’s nutrient solution alone; WW+ML: half-strength Hoagland’s nutrient solution and 100 μmol/L melatonin by leaf; WW+MR: half-strength Hoagland’s nutrient solution and 100 μmol/L melatonin by root; PEG: half-strength Hoagland’s nutrient solution plus 15% PEG 6000 treatment; PEG+ML: half-strength Hoagland’s nutrient solution plus 15% PEG 6000 treatment and 100 μmol/L melatonin by leaf; PEG+MR: half-strength Hoagland’s nutrient solution plus 15% PEG 6000 treatment and 100 μmol/L melatonin by root. PH: plant height; SP: seed pod; SPP: seeds per plant; SW: seed weight. The data represent the mean ± SE of five replicate samples. Different lowercase letters indicate significant differences according to Duncan’s multiple range test (*P*<0.05).

### Principal component analysis

**Fig 7:**
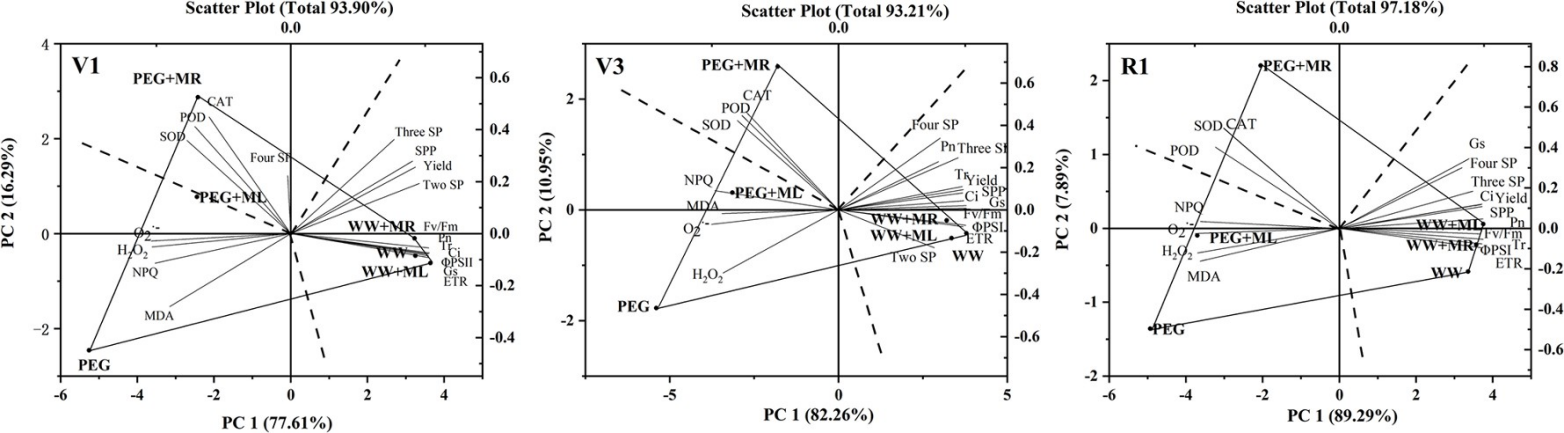
Analysis of all indexes with different treatment by GGE biplot. WW: half-strength Hoagland’s nutrient solution alone; WW+ML: half-strength Hoagland’s nutrient solution and 100 μmol/L melatonin by leaf; WW+MR: half-strength Hoagland’s nutrient solution and 100 μmol/L melatonin by root; PEG: half-strength Hoagland’s nutrient solution plus 15% PEG 6000 treatment; PEG+ML: half-strength Hoagland’s nutrient solution plus 15% PEG 6000 treatment and 100 μmol/L melatonin by leaf; PEG+MR: half-strength Hoagland’s nutrient solution plus 15% PEG 6000 treatment and 100 μmol/L melatonin by root. PH: plant height; SP: seed pod; SPP: seeds per plant; SOD: superoxide dismutase; POD: peroxidase; CAT: catalase; MDA: the content of malondialdehyde; H_2_O_2:_ hydrogen peroxide content; O_2_^·-^: superoxide anion production raes; Fv/Fm: energy conversion efficiency; NPQ: photochemical quenching coefficient; Φ_PSII_: PSII actual photochemical quantum yield; ETR: electron transfer rate; Pn: net photosynthetic rate; Ci: intercellular CO_2_ concentration; Tr: transpiration rate; Gs: stomatal conductance.

According to the information from the GGEbiplot (Fig 7) in stages V1, V2 and R1, PC1 was explained 77.61%, 82.26% and 89.29% of the variation, and PC2 was 16.29%, 10.95% and 7.89%, showing that 93.89%, 93.21% and 97.18% of all true information was processed and that the analysis results were highly reliable.

A polygon was formed by connecting the outermost treatment points (PEG, PEG+MR, WW+MR, WW+ML, WW) with straight lines. The three dotted lines perpendicular to each side from the center point divide the entire figure into three areas. All indicators are distributed among the three regions. We can see that four indicators NPQ, O_2_^·−^, MDA, and H_2_O_2_ are divided into PEG treatment areas in stages V1, V2 and R1. Therefore, the four indicators had the greatest influence under water deficit stress, which is also reflected in the previous analysis. In addition, the angle between these four indicators was less than 90°, indicating a significant positive correlation between them. Moreover, the angle between H_2_O_2_ and O_2_^·−^ was relatively small, showing that the correlation between the two was relatively strong. Therefore, it can be preliminarily concluded that the content of NPQ, O_2_^·−^, MDA, and H_2_O_2_ of soybean leaves under PEG treatment were relatively high; thus, the degree of damage was greater than that in the other treatments.

CAT, POD and SOD were distributed in the region in which the PEG+MR treatment was the apex, indicating that the three indicators were highest under PEG+MR treatment. Those indicators were a positive correlation, because the angle is less than 90°. In addition, the angle between antioxidant enzyme activity (CAT, POD and SOD) and ROS (H_2_O_2_ and O_2_^·−^) is also less than 90°, indicating that ROS accumulation under PEG+MR treatment induced antioxidant enzyme activity and increased osmotic adjustment substances. PEG stress led to an increase in the activity of antioxidant enzymes and osmotic adjustment substances, and the activity and content of the two substances further increased in response to melatonin by root irrigation to remove excess H_2_O_2_ and O_2_^·−^, regulate cell osmotic potential, and alleviate cell damage, all of which are essentially consistent with previous findings (Figs 2–4). These results reflect the regulatory effect of melatonin on the antioxidant enzyme activity and osmosis-regulating contents under PEG stress.

Other indicators such as fluorescence characteristics, gas exchange parameters, two sp, three sp and yield were distributed within the well-watered region. These results showed that, compared with the other treatments, the well-watered treatment had a significant effect on the fluorescence characteristics, gas exchange parameters and yield of soybean, all of which are essentially consistent with previous findings (Figs 5 and 6, Table 3). Moreover, the angle between the fluorescence characteristics (Fv/Fm, ΦPS (II) and ETR) and gas exchange parameters (Pn, Gs, Ci and Tr) was less than 90°, indicating a positive correlation.

## Discussion

Water-deficit stress is an important environmental factor inhibiting plant growth and development [50], and it is closely related to plant growth and development. PEG 6000 at a concentration of 15% had a particularly prominent inhibitory effect on soybean growth (Table 1). PEG-induced water deficit has an adverse effect on the physiological characteristics of soybean [51]. However, the application of exogenous substances can alleviate PEG-induced water deficit stress [52], and the effect varies for some physiological characteristics of soybean [53].

Drought results in a decrease in the leaf area index of soybean [54] and inhibits increases in the stem weight and root weight of soybean [55]. In our experiment, compared with the WW condition, PEG 6000 treatment simulated water deficit stress and significantly reduced the LAI, LAD and accumulation of dry matter in soybean seedlings and flowering stages. Compared with PEG alone, the application of exogenous melatonin under water deficit stress, improved the LAI and LAD. In addition, the accumulation of dry matter in roots, stems and leaves were also increased, possibly because melatonin maintained a high photosynthetic rate in the plant leaves, perhaps in particular by improving the efficiency of photosystem II (Fv/Fm). Ren [56] described the soaking of soybean seeds with melatonin that were sowed in an experimental field and then harvested at the end of a three-week growing period. The application of exogenous melatonin significantly increased the number of nodules and shoot biomass. Our research indicated that in stage V1, dry matter accumulation in different organs did not differ under water deficit stress between plants that were treated with melatonin and plants that were not treated, while the dry matter accumulation in leaves and roots in stage V3 and leaves in stage R1 was significantly higher in treated than in nontreated plants. This finding suggests that melatonin under water deficit stress has no alleviating effect on soybean morphogenesis in stage V1, but a significant effect in stage V3 and R1. This difference is possibly because the nutrients needed for organ building in stage V1 mainly came from seeds, and therefore, exogenous additives had little influence on the dry matter accumulation of soybean in this stage; in contrast, in stages V3 and R1, exogenous additives had a significant influence on the dry matter accumulation in soybean leaves and roots, consistent with the results of a study [57] on soybean nutrient requirements.

Inhibition of photosynthesis is one of the main hazards of water deficit stress [58]. Water deficit may limit photosynthesis by inducing stomatal closure (stomatal restriction), damaging the metabolic process (metabolic restriction) or both [59]. The consistent trends among intercellular carbon dioxide concentrations (Ci), net photosynthetic rates (Pn) and stomatal conductance (Gs) indicated that the number of CO_2_ molecules in plant cells decreased to influence the effectiveness of CO_2_ in the plant body. The photosynthetic efficiency of the crop plant was affected by changes in carbon metabolic approaches in the leaves, suggesting that the prevailing influencing factor in photosynthesis in this study was stomatal. In contrast, the prevailing factors in other studies have been nonstomatal [60–61]. The results of the present study show that during water deficit stress, the Gs of soybean leaves was significantly reduced, and the stomatal conductance decreased such that the leaf Tr and Ci decreased. CO_2_ is an important raw material for photosynthesis. Reductions of photosynthetic raw materials lead to declines in the Pn in soybean leaves, partly due to of water stress-induced stomatal closure. Stomatal restriction is generally considered a short-term response to water stress that is mediated by regulation or closure of stomata, while nonstomatal effects are generally considered to involve damage to photosynthetic cells or organs due to long-term water stress [62–63]. In this experiment, the Gs, Tr and Ci were improved in soybean leaves under exogenous melatonin treatment with water deficit stress compared with water deficit stress alone, as was Pn (Fig 2); these findings indicated that exogenous melatonin could regulate Gs in soybean leaves under water deficit stress to improve the Ci, increase the availability of raw materials for photosynthesis to increase the Pn, alleviate the negative effect of stomatal closure on the net photosynthesis rate under water deficit stress, and prevent irreversible damage to photosynthetic organs. Therefore, it is inferred that the factors influencing the decrease in the net photosynthetic rate under water deficit stress in this study were stomatal. Under water stress, plants accumulate the signaling hormone abscisic acid, causing rapid stomatal closure to reduce water loss, which is a short-term adaptation to water stress. In addition, the results of the gas exchange parameter test in this study also proved that PEG 6000 at a concentration of 15% did not cause serious damage to photosynthetic organs in a short period of time; furthermore, short-term damage caused by water deficit stress to the photosynthetic organs of plant leaves could be alleviated by exogenous melatonin.

Water stress directly affects the structure and activity of photosynthetic organs in plant leaves as well as the photochemical reactions and dark reactions of photosynthesis [64]. Therefore, chlorophyll fluorescence parameters are very sensitive to water deficit stress and can reflect the absorption, transmission and conversion of light energy in leaves [65]. Carcia et al. [66] suggested that water stress has no effect on Fv/Fm. In contrast, in our experiment, the Fv/Fm of soybean under water stress decreased to different degrees (Fig 3). Zhou et al. [67] indicated that the Fv/Fm reflects the health of the PSII system, suggesting that water stress affects the initial PSII light energy conversion rate and potentially affects PSII activity in soybean leaves to reduce photosynthesis. Φ_PS(II)_ is the actual photosynthetic efficiency of PSII [68]. In this study, comparison with gas exchange parameters suggested a positive correlation between Φ_PS(II)_ and net photosynthetic efficiency (Pn) to a certain extent. Φ_PS(II)_ decreases mainly because of the photoelectric transfer rate (ETR) [69]. In this experiment, a significant reduction in ETR occurred under water stress, reducing the energy distribution and electron transfer between PSI and PSII in soybean leaves and further reducing the effectiveness of the plants to absorb light energy to influence soybean photosynthesis. NPQ reflects the heat consumption capacity and apparent light protection function of plants and can protect the photosynthetic organs of soybean seedlings [70]. In this study, water stress treatment significantly increased NPQ in soybean leaves, showing that under adverse conditions, soybean seedling consume light energy in the form of heat to reduce irreversible damage to soybean leaves’ PSII systems. At present, there are many research reports about exogenous hormone alleviation of plant damage under adverse conditions, such as high temperatures in rice [71] and drought in corn [72] and apple [73]. Studies have shown that under water stress conditions, exogenous hormones improve the Fv/Fm, Φ_PS(II)_ and ETR and reduce NPQ, consistent with the results of our research. In this experiment, the application of exogenous melatonin under water stress improved the Fv/Fm, ETR and Φ_PS(II)_ in soybean leaves, enhanced the photosynthetic electron conduction efficiency of soybean leaves under water stress, and improved the capability of leaves to capture light energy. NPQ was significantly reduced. These findings suggest that exogenous melatonin enhances the electron capture efficiency in the reaction centers of soybean leaves and maintains a high electron transfer rate. With these changes, absorbed light energy can be better used for photosynthetic electron transfer, reducing excess light energy dissipation in the form of heat, enhancing photorespiration, and protecting photosynthetic mechanisms.

Many studies have shown that water stress can lead to the disturbance of antioxidant enzyme activity in plants [74]. Excessive ROS generated under water stress significantly increase MDA levels and osmotic conductivity in plant cells, and ROS levels are considered markers of the degree of membrane lipid peroxidation [75]. Membrane lipid peroxidation in biological membranes impairs membrane function, reduces fluidity, and reduces membrane binding receptor and enzyme activity [76]. In this study, antioxidant enzyme activity and MDA levels in soybean leaves were increased under water stress, indicating that excessive ROS production was induced under water stress, and the accumulation of MDA increased the degree of membrane lipid peroxidation (Fig 2), consistent with the results of previous studies on soybean and corn [77]. Under water stress, plant cells initiate various stress responses to cope with the effects of water shortage. Melatonin is a high-intensity free radical scavenger with spectral properties that can easily pass through the cell membrane and enter the cell body to fundamentally maintain the antioxidant activity of a cell [77]. Therefore, melatonin can directly scavenge excess ROS [78], and it can also promote the accumulation of other antioxidants and antioxidant enzymes to indirectly remove ROS [79]. In our study, the application of exogenous melatonin under water stress significantly increased the activity of antioxidant enzymes, reduced the accumulation of MDA, and decreased the content of ROS (H_2_O_2_ and O_2_^·-^) in leaves (Fig 3), thus regulating the oxidative balance of soybean, maintaining intracellular ROS concentrations, protecting against ROS accumulation, and reducing the damage caused by water stress on membrane lipid peroxidation. The application of melatonin by root irrigation generated the best effect.

In addition, the application of exogenous melatonin increased the plant height, pod numbers and total grain numbers of soybean under water stress to increase the soybean yield under water stress, consistent with the results of a study in which the seeds were coated with melatonin [80]. The yield was slightly higher in stage V1 than stages V3 and R1, indicating that the effect of water stress on soybean yield was greater in stages V3 and R1 than stage V1; the reason for this difference may be that nutrients in vegetative organs in stage V1 derive from seeds, while exogenous substances have no effect on the morphogenesis of vegetative organs. The applied melatonin entered the soybean plant to maintain the oxidative balance in cells and removed excessive ROS to better alleviate the impact of external water stress on soybean in the V1 stage compared with the V3 and R1 stages. Nutrients in the V3 and R1 stages are absorbed from the external environment and obtained through nitrogen fixation during root nodulation; these processes are greatly affected by external water stress, thus affecting soybean morphogenesis and significantly reducing the soybean yield in V3 and R1 compared with V1, consistent with the results of previous studies with Ren [56] in which the seeds were soaked in melatonin. Based on the results, it can conclude that the application of melatonin by root irrigation contributes more to the improvement of soybean yield than application by foliar spraying.

The results show that melatonin increases plant growth, seed production, and abiotic stress tolerance in soybean plants, possibly through the enhancement of photosynthesis, carbohydrate metabolism, and antioxidative actions. These discoveries provide a basis for the potential application of exogenous melatonin in increasing soybean production and warrant further studies to dissect the molecular mechanism(s) that regulate melatonin-mediated water-deficient stress in soybean plants.

## Conclusion

In summary, the application of exogenous melatonin under water stress in the V1 and V3 stages of soybean increases the leaf area index and leaf area duration, increases the dry matter accumulation, enhances antioxidant enzyme (SOD, POD, and CAT) activity, reduces ROS (H_2_O_2_ and O_2_^·-^) levels, reduces MDA content, improves the Fv/Fm, ETR, Φ_PS (II),_ NPQ and other chlorophyll fluorescence characteristics, and improves the photosynthetic rate in soybean leaves to enhance photosynthesis and ultimately improve the soybean yield under water stress. Application of melatonin by root irrigation is better than application by foliar spraying. This is the first report of the application of exogenous melatonin in soybean seedlings and flowering stages during induced drought stress and the first analysis to use an experimental approach investigating leaf chloroplast morphology and photosynthesis.

## Supporting information

S1 File Supporting information data. This file contains date including antioxidant activity, MDA levels, H_2_O_2_ contents, O_2_^·-^ production rates, fluorescence characteristics, gas exchange parameters, principal component analysis data.
(XLSX)
